# Risk factors of retinopathy in type 2 diabetes mellitus at a tertiary care hospital, Bahawalpur Pakistan.

**DOI:** 10.12669/pjms.292.3066

**Published:** 2013-04

**Authors:** Sadiq Hussain, Muhammad Rashad Qamar, Muhammad Arshad Iqbal, Ameer Ahmad, Ehsan Ullah

**Affiliations:** 1 Sadiq Hussain, Department of Pathology, Quaid-e-Azam Medical College (QAMC), Bahawalpur, Pakistan.; 2 Muhammad Rashad Qamar, Department of Ophthalmology, Quaid-e-Azam Medical College (QAMC), Bahawalpur, Pakistan.; 3 Muhammad Arshad Iqbal, Department of Pathology, Quaid-e-Azam Medical College (QAMC), Bahawalpur, Pakistan.; 4 Ameer Ahmad, Department of Paediatrics, Quaid-e-Azam Medical College (QAMC), Bahawalpur, Pakistan.; 5 Ehsan Ullah, Department of Pathology, Quaid-e-Azam Medical College (QAMC), Bahawalpur, Pakistan.

**Keywords:** Type 2 diabetes mellitus, Diabetic retinopathy, HbA1c, Cholesterol, Microalbuminuria

## Abstract

***Objectives:*** To find out the risk factors of diabetic retinopathy in type 2 diabetes mellitus.

***Methodology:*** It was a cross-sectional study involving 300 patients of type 2 diabetes. Clinical history, relevant examination including fundoscopy and lab investigations were done. Data was analysed with SPSS 17.0. T-test and chi square/Fischer exact were applied to determine significance.

***Results:*** Mean age of the patients was 49.04 ± 0.69 years with slight female predominance with male to female ratio of 3:4. Average duration of disease was 7.17 ± 0.38 years. Diabetic retinopathy was diagnosed in (74, 23.9%). Mean HbA1c was 8.15% in patients with retinopathy and 8.884% in those who had no retinopathy (p=0.08). However, duration of DM, age of patients, male gender, high total cholesterol, high LDL and microalbuminuria were significantly associated with the development of retinopathy.

***Conclusions:*** Diabetic retinopathy was found in 23.9% of type 2 diabetics. It was associated with duration of disease, age at presentation, male gender, high total cholesterol, high LDL and microalbuminuria. A single high level of HbA1c was not associated with retinopathy.

## INTRODUCTION

Type 2 diabetes mellitus (DM) is a group of disorders characterized by hyperglycemia and associated micro vascular (retinal, renal, possibly neuropathic), macro vascular (coronary, peripheral vascular), and neuropathic (autonomic, peripheral) complications. Unlike Type 1 diabetics, patients are not absolutely dependent upon insulin for life, even though many of these patients are ultimately treated with insulin.^1^

Prevalence of type 2 diabetes and diabetic Retinopathy in Pakistan is 10% and 27% respectively.^2^^,^^3^ A spectrum of retinal changes accompanying long-standing diabetes may advance to cause the abnormal retinal neovascularization, which may lead to severe visual loss and even blindness. Diabetic retinopathy is a leading cause of visual impairment among diabetic patients.

There are many reports from various countries about incidence and prevalence of diabetic retinopathy and the risk factors associated with this condition. Limited studies are available on risk factors associated with diabetic retinopathy in Pakistan. Stratton et al reported that older age, male gender, hyperglycemia (persistently raised HbA1c), hypertension and smoking were significantly associated with the incidence and progression of retinopathy in type 2 diabetic patients.^4^ Chatziralli IP et al found duration of diabetes, hypertension, poor glycemic control and male gender as strong independent risk factors of retinopathy and they reported that the age was a confounding factor.^5^ A study on large number of diabetics having retinopathy (n=7577) from Asia found duration of disease, elevated blood glucose levels and high blood pressure as independent risk factors.^6^

In the blood stream are the red blood cells, which contain a molecule called hemoglobin. Glucose sticks to the hemoglobin to make a ‘glycosylated hemoglobin’ molecule, known as HbA1c. More the glucose and more HbA1C will be present in the blood. Red cells remain alive for 8-12 weeks before they are replaced. By measuring the HbA1c, how high blood glucose has been on average over the last 8-12 weeks can be assessed. A normal non-diabetic HbA1C is 3.5-5.5%. The HbA1c test is currently one of the best ways to check the control of diabetes. Persistent elevation in serum glucose (HbA_1c_) increases the risk for the long-term vascular complications of diabetes such as retinopathy.^7^^,^^8^

High levels of hemoglobin A1c (HbA1c) for long duration are important risk factor for progression to high risk proliferative diabetic retinopathy and decreased visual acuity. Intensive glycemic control for long duration (HbA1c levels normal or near normal) reduces the risk of retinopathy significantly. Intensive therapy is most effective when initiated early in the course of the diabetes, demonstrating a beneficial effect over the course and progression of retinopathy.^9^^,^^10^

Our objective was to find out various risk factors associated with retinopathy in type 2 diabetes mellitus.

***Operational Definitions:***

***Diabetic retinopathy*** A diagnosis of diabetic retinopathy will be established if the subject has a minimum of one micro aneurysm in any field, or showing hemorrhages (dot & blot, or flame shaped), or maculopathy (with or without clinically significant edema).

***High glycosylated hemoglobin (HbA1c)*** Value of glycosylated hemoglobin more than 7% will be taken as high.

***Poor:*** A person who earn less than 2$ per day.

## METHODOLOGY

Patients of type 2 diabetes mellitus (DM) of both genders irrespective of duration of diabetes, more than 30 years of age attending Bahawal Victoria Hospital, Bahawalpur were included in this cross sectional descriptive study. It was conducted at Department of Pathology, Department of Ophthalmology Bahawal Victoria Hospital/ Quaid-e-Azam Medical College Bahawalpur between July 2011 to June 2012.Patients of type I DM, Known cases of retinopathy which may be due to causes other than DM and value of HbA1c less than 7% were excluded.

***Sample size:*** Sample size was 299, rounding off the nearest whole number 300. The prevalence of retinopathy in type2 DM was 27%. Purposive Sampling technique was used for this study. The study was approved by the local ethical committee. After taking written and verbal consent from the patients, a structured proforma containing demographic features, duration of DM, oral or insulin therapy was filled. Blood samples were collected for analysis of HbA1c, serum total cholesterol and LDL cholesterol. HbA1c was measured by Boronate affinity chromatography (Clover A1c). Total and LDL cholesterol were measured by automated chemistry analyzer. A detailed fundus examination was performed by slit lamp bio microscopy.

***Data analysis:*** Data was analyzed using SPSS version 17. The results were evaluated using frequencies, proportions and group means. The frequencies and percentages were calculated for all the qualitative data including gender, age group, and diabetic retinopathy.

## RESULTS

Mean age of the patients was 49.04 ± 0.69 years. Females were slightly predominant (n=174, 56.1%) as compared to males (M:F ratio 3:4). All patients were type 2 diabetics with average duration of disease of 7.17 ± 0.38 years. Diabetic retinopathy was diagnosed in about 1/4^th^ patients (n=74, 23.9%). Mean HbA1c was 8.71 ± 0.08 percent. A majority of patients was literate (n=198, 63.9%) as compared to illiterate (n=112, 36.1%), similarly 187 patients (60.3%) had good socioeconomic status as compared to poor patients (n=123, 39.7%). It was found that patients (n=105, 33.9%) were counseled by the treating doctor about compliance to medication and complications of diabetes mellitus if poorly controlled, as compared to 205 (66.1%) patients who were just prescribed treatment without counseling. Family cooperation was noted in 227 (73.2%) patients. History of Hakeem medication was found in 119 (38.4%) patients. As shown in Table-I, there was no statistical difference of mean HbA1c among the patients who had diabetic retinopathy or not (p = 0.080).

**Table-I:** Comparison of HbA1c in diabetic patients having retinopathy

	*Retinopathy*	*N*	*Mean*	*Std. Deviation*	*Std. Error Mean*
*HbA1c*	Yes	74	8.154%	1.1169	.1298
	No	236	8.884%	1.4488	.0943

 Mean duration of diabetes mellitus who had developed retinopathy was 16.05 ± 0.903 years as compared to 4.39 ± 0.197 years who had not developed retinopathy yet. Independent Samples t Test was applied and a statistically significant difference (0.0001) was calculated as shown in Table-II.

**Table-II:** Association of duration of diabetes with development of retinopathy

	*N*	*Mean duration in years*	*Std. Deviation*	*Std. Error Mean*
*Retinopathy*	74	16.05	7.767	.903
*No Retinopathy*	236	4.39	3.029	.197

.Although there was slight female preponderance among type 2 diabetics however, males were found to have higher frequency of retinopathy (n=41/136, 30.15%) as compared to females (n=33/174, 18.96%). Similarly, high serum total cholesterol, high serum LDL-cholesterol, presence of albuminuria and insulin therapy were found to have significant associations with the development of diabetic retinopathy as shown in Table-III. Chi square was applied which revealed significant difference (p = 0.023) between the two genders.

**Table-III:** Association of various risk factors with diabetic retinopathy

*Risk factors*	*Retinopathy*		*p value*
	*Frequency*	*Percentage*	
Gender Male Female	41/136 33/174	30.15% 18.96%	0.023
Age ≤49 years >49 years	13/164 61/146	7.93% 41.78%	0.0001
Total cholesterol Normal High	19/119 55/191	15.97% 28.79%	0.010
LDL cholesterol Normal High	33/198 41/112	16.67% 36.61%	0.0001
Albuminuria Absent Present	14/193 60/117	7.25% 51.28%	0.0001
Therapy Insulin Oral hypoglycemics	49/74 25/234	66.22% 10.68%	0.001

## DISCUSSION

Diabetes mellitus is a global problem and diabetic retinopathy is a common complication of this systemic disorder with its overall global prevalence of 34.6% (range = 17.99% – 51.2%).^11^ However, the studies from within Pakistan show the prevalence of diabetic retinopathy ranging from 9.0% to 43.0%.^12^^-^^15^ Insulin treatment, duration of diabetes, age at examination, HbA1c, systolic blood pressure, cholesterol, triglyceride and microalbumin were found to be significantly related to the development and the progression of retinopathy among type 2 diabetics from Kuwait by AlKharji et al.^16^ However, a recent study by Chitrazalli and colleague documented that effect of age is a confounding factor but years since DM diagnosis, hypertension, serial high HbA1c levels and male sex were independent risk factors for the development of diabetic retinopathy.

In our study there is no significant association of retinopathy with single high HbA1c level among patients of type 2 diabetes. It is common observation that most of the patients of type 2 diabetes have high HbA1c levels at the time of diagnosis of the disease but they have no retinopathy at that time. There are many studies conducted in various countries over the period of last decade or so which provide fair evidence of association of persistently high HbA1c with the development of various grades of retinopathy in type 2 diabetic patients.^17^^-^^19^

In our patients, the overall frequency of retinopathy was 23.9% whereas a study from Karachi involving large number of diabetic patients reported similar frequency of retinopathy (27.4%).^20^ Another study conducted recently, showed higher frequency (34.5%),^21^ however this difference may be attributed to patient selection in that study and/or other demographic variables.

Our study showed that the retinopathy was found in 74 patients whose average duration of type 2 diabetes was 16.05±0.90 years as compared to those who had no retinopathy. The latter group of patients had 236 patients with mean duration of disease of 4.39±0.20 years. Thus a strong correlation was found between the retinopathy and duration of diabetes. Many other studies have also identified the duration of diabetes as the major risk factor of the development of diabetic retinopathy.^22^ Shaukat et al also showed positive correlation between the duration of diabetes with the incidence of retinopathy as in current study.^23^

## CONCLUSIONS

Diabetic retinopathy was found in 23.9% of type 2 diabetics which was significantly associated with male gender, duration of diabetes, older age, high cholesterol, high LDL, albuminuria and insulin therapy. No association of high HbA1c levels for short duration (months not years) was found with diabetic retinopathy. Persistently raised HbA1c level may be associated with diabetic retinopathy.

## Author’s Contribution:

**SH and MRQ **formulated the study design and helped in the final drafting of the manuscript.

**MAI and AA **critically revised the manuscript.

**EU **has performed the data analysis, interpretation, drafted the manuscript, and substantially contributed to conception and design of the study.

## References

[1] American Diabetes Association (2011). Standards of medical care in diabetes. Diabetes Care.

[2] Qayyum A, Amir Babar AM, Das G (2010). Prevalence of diabetic retionpathy in Quetta, Balochistan. Pak J Ophthalmol.

[3] Mazhar PS, Awan MZ, Manzar N (2010). Prevalence of type-II diabetes and diabetic retinopathy. J Coll Phys Surg Pak.

[4] Stratton IM, Kohner EM, Aldington SJ (2001). UKPDS 50: Risk factors for incidence and progression of retinopathy in type 2 diabetes over 6 years from diagnosis. Diabetologia.

[5] Chatziralli IP, Sergentanis TN, Keryttopoulos P (2010). Risk factors associated with diabetic retinopathy in patients with diabetes mellitus type 2. BMC Research Notes.

[6] Wang FH, Liang YB, Peng XY (2011). Risk factors for diabetic retinopathy in a rural Chinese population with type 2 diabetes: the Handan Eye Study. Acta Ophthalmologica.

[7] Cohen RM, Smith EP (2008). Frequency of HbA1c discordance in estimating blood glucose control. Curr Opin Clin Nut Metab Care.

[8] Tapp RJ, Tikellis G, Wong TY, Harper CA, Zimmet PZ (2008). Longitudinal association of glucose metabolism with retinopathy: results from the Australian Diabetes Obesity and Lifestyle study. Diabetes Care.

[9] American Diabetes Association (2010). Diagnosis and classification of diabetes mellitus. Diabetes Care.

[10] Rodriquez M, Kerrison JB, Alfaro DV (2009). Metabolic control and diabetic retinopathy. Curr Diabetes Rev.

[11] Jau JWY, Rogers SL, Kawasaki R (2012). Global prevalence and major risk factors of diabetic retinopathy. Diabetes Care.

[12] Shaikh MA, Gillani S, Yakta D (2010). Frequency of diabetic retinopathy in patients after ten years of diagnosis of type 2 diabetes mellitus. J Ayub Med Coll Abbottabad.

[13] Iqbal T, Zafar J (2009). Frequency of retinopathy in newly diagnosed type 2 diabetes mellitus. Rawal Med J.

[14] Shera AS, Jawad F, Maqsood A, Jamal S, Azfar M, Ahmed U (2004). Prevalence of chronic complications and associated factors in Type 2 Diabetes. J Pak Med Assoc.

[15] Wahab S, Mahmood N, Shaikh Z, Kazmi WH (2008). Frequency of retinopathy in newly diagnosed type 2 diabetes patients. J Pak Med Assoc.

[16] AlKharji F, Alshemmeri N, Mehrabi L (2006). Prevalence and risk factors for diabetic retinopathy among Kuwaiti diabetics. Kuwait Med J.

[17] Shichiri M, Kishikawa H (2000). Long-term results of the Kumamoto Study on optimal diabetes control in type 2 diabetic patients. Diabetes Care.

[18] Salti H, Cavallerano JD, Salti N, Jawhari DJ (2011). Nonmydriatic Retinal Image Review at Time of Endocrinology Visit Results in Short-Term HbA1c Reduction in Poorly Controlled Patients with Diabetic Retinopathy. Telemedicine and e-Health.

[19] Chorny A, Lifshits T, Kratz A, Levy J, Golfarb D, Zlotnik A, Knyazer B (2011). Prevalence and risk factors for diabetic retinopathy in type 2 diabetes patients in Jewish and Bedouin populations in southern Israel. Harefuah.

[20] Mahar PS, Awan MZ, Manzar N, Memon MS (2010). Prevalence of Type-II Diabetes Mellitus and Diabetic Retinopathy: The Gaddap Study. J Coll Phys Surg Pak.

[21] Zheng Y, Lamoureux EL, Lavanya R (2012). Prevalence and Risk Factors of Diabetic Retinopathy in Migrant Indians in an Urbanized Society in Asia. Ophthalmology.

[22] Takao T, Ide T, Yanagisawa H (2011). The effect of fasting plasma glucose variability and time-dependent glycemic control on the long-term risk of retinopathy in type 2 diabetic patients. Diab Res Clin Prac.

[23] Shaukat A, Arain TM, Ali I, Alam MF, Sajid MA, Nasreen S (2011). Frequency of retinopathy among adult diabetics in Bahawalpur City. Pak J Pathol.

